# Clinical genetics: past, present and future

**DOI:** 10.1038/s41431-022-01041-w

**Published:** 2022-01-25

**Authors:** Eva Tromans, Julian Barwell

**Affiliations:** 1 Department of Clinical Genetics, University Hospitals of Leicester NHS Trust, Leicester Royal Infirmary, Leicester, LE1 5WW UK; 2 Department of Genetics and Genome Biology, University of Leicester, Leicester, LE1 7 RH UK

**Keywords:** Health policy, Health care economics, Genetic counselling, Genetic testing

Medicine is a constantly evolving field, with specialities developing and declining with the advancement of technology and knowledge. Clinical Pharmacology, previously a mainstream speciality, is now rarely recognised as a separate subspecialty within medicine. With the development of computer sciences and Artificial Intelligence, many question the future role of clinicians in specialties such as plain-film Radiology [1].

Technological improvements in Clinical Genetics have progressed apace, transforming the landscape of both diagnosis and management of inherited genetic diseases. Initially testing for single gene nucleotide changes, it is now possible to screen larger panels and exomes. The drop in prices from ~US$3 billion to sequence the first human genome, to current prices of ~US$1000 per genome means that even whole-genome sequencing can be undertaken more routinely [2, 3]. This has led towards mainstreaming of Genomic Services. Conversely, it has meant that the key roles of Clinical Geneticists, including gatekeeping of tests, identification of risk and variant interpretation, will likely change.

## The past: gatekeeping

In the past, diagnostic laboratory tests to establish the cause of inherited diseases were expensive, slow and limited. Turnaround times involved months from starting a consultation to patients receiving a diagnosis. This meant that the clinician’s skill in taking a thorough history and recognising dysmorphological features were key to identifying the relevant individuals for testing. Patients were only tested after a thorough assessment by a Clinical Geneticist, who therefore acted as a gatekeeper for expensive tests. Even after individuals were correctly confirmed to have specific conditions, in many cases, options to significantly alter outcomes were either limited, or involved life-changing surgery.

## The present: expansion and transformation

As prices dropped, availability of testing increased, and the 100,000 Genomes Project established infrastructure, enabling further expansion. This increased testing drove laboratory-based improvements, including sample logistics to allow for higher throughput, in addition to standardisation of reporting, paving the way for the development of the nationalized Genomic Medicine Service [4].

As our knowledge of certain conditions and treatment options improved, this changed the dynamic regarding who can order tests (democratisation) and wider impacts of testing (additional and incidental findings) with a reduced role for gatekeeping.

This has been aided by the development of the National Genomic Test Directory for Rare and Inherited Disease [5], an annually updated list of test panels that are most appropriate to utilise for the diagnosis of specific suspected conditions. Such accessibility led to improvements in patient management, reducing turnaround times, and negating the need for subsequent specialist referral.

However, full adjustment to these changes will involve significant adaptations from both mainstream medical specialities and Clinical Genetics. Education needs to be upscaled for subspecialties to be trained in understanding the risks and impacts of the tests they are ordering, and how to counsel patients appropriately.

Advances in genetic technology have increased the option of high-throughput testing, including whole-genome testing in trios (parents and affected child to identify de novo and recessive traits) and matched tumour and blood testing (for acquired mutations), the latter of which is becoming increasingly routine [2]. This influx of data and linking these to predictions of risk for populations has placed heavy demands on bioinformatics analysis, including both software and manual assessment, to aid interpretation of the resulting information.

Additionally, increased quantities of data result in an increased number of variant identification, often of unclear significance. Standardisation, such as in the guidelines set out by American College of Medical Genetics and Genomics, which uses a point-based system to assess likelihood of pathogenicity of a variant, has attempted to address this issue [6]. This could lead to a new role for Clinical Geneticists, offering clinical support, and aiding in reverse phenotyping, whereby an individual has investigations prior to a dysmorphological review. In complex cases, Geneticists may also be required to assist with determining the right test (read depth/trinucleotide repeats/methylation analysis) to reach the correct diagnosis.

This increased amount of data leaves questions regarding data ownership and confidentiality. This is a topical subject for all specialities, as the move towards digitisation of healthcare records progresses. It presents certain challenges, including possible genetic discrimination, but the aim is to implement these changes with a focus on patient and public engagement with regards to discussions around data sharing, privacy, insurance and healthcare rationing. This requires a clear consent process, which is being standardised through the introduction of the record of discussion documents and hopefully in time, the development of the National Genomic Information System [7].

## The future: mainstreaming

The potential impacts of these changes suggest that Clinical Genetics needs to reflect on its position if it is no longer to focus on gatekeeping tests or interpreting results, especially as society is changing its view over autonomy for genetic and predictive testing. The field remains vital in driving the technological advances in pharmacogenetics and precision medicine to improve our understanding in predicting individual responses to medical interventions, as outlined by Dame Sally Davies in her 2016 report [8]. The number of genetic associate roles may also need to increase, as mainstreaming teams may struggle with the expansion of their clinical responsibilities and the corresponding administrative work required for data entry.

As risk stratification moves towards computerised systems, questions remain regarding exactly who will take responsibility for these decisions, and how this may affect clinical autonomy. An increasing reliance on computers could paradoxically place healthcare delivery at risk of actually depersonalising healthcare, if it is being delivered by automated software on machines without a sense of moral judgement; unable to weigh up complex social and emotional factors.

With changes in diagnostic tests, variant interpretation, predictive testing, counselling dynamics, risk assessment algorithms and the development of online patient-led care, navigation aids for gene carriers means that redefining the role of Clinical Geneticists may well be required.

Apart from identifying molecular causes for disease, predicting risk and response in families, populations and individuals, there will be new logistic, educational and time-related challenges for mainstream specialties to take on board all aspects of these new roles within a strong governance framework.
